# Functional Properties of Cancer Epithelium and Stroma-Derived Exosomes in Head and Neck Squamous Cell Carcinoma

**DOI:** 10.3390/life12050757

**Published:** 2022-05-20

**Authors:** Yang Li, Shengtao Gao, Qi Hu, Fanglong Wu

**Affiliations:** 1 Department of Oral Pathology, College of Stomatology, Ningxia Medical University, South Sheng Li Street 804, Yinchuan 750004, China; liyang@nxmu.edu.cn; 2 Key Laboratory of Stomatology of Fujian Province, School and Hospital of Stomatology, Fujian Medical University, Yang Qiao Middle Road 246, Fuzhou 350004, China; 3 State Key Laboratory of Oral Diseases, West China College of Stomatology, Sichuan University, South Renmin Road, Sec. 3, No. 14, Chengdu 610041, China; 2019151640119@scu.edu.cn; 4 College of Public Health and Management, Ningxia Medical University, South Sheng Li Street 1160, Yinchuan 750004, China; huqi@nxmu.edu.cn; 5 State Key Laboratory of Oral Diseases, National Center of Stomatology, National Clinical Research Center for Oral Diseases, Frontier Innovation Center for Dental Medicine Plus, West China Hospital of Stomatology, Sichuan University, South Renmin Road, Sec. 3, No. 14, Chengdu 610041, China

**Keywords:** head and neck squamous cell carcinoma, exosomes, applications, biomarkers, antitumor therapy

## Abstract

Stroma–cancer cell crosstalk involves a complex signaling network that contributes to tumor progression, including carcinogenesis, angiogenesis, migration, invasion, and therapy resistance in cancers. Exosomes, as extracellular membranous nanovesicles released by almost all types of cells, including tumor cells and stromal cells, play a critical role in signal delivery and material communication, in which the characteristics of their parent cells are reflected. The tumor or stroma-derived exosomes mediate cell–cell communication in the tumor microenvironment by transporting DNA, RNA, proteins, lipids, and metabolites. Recent studies on head and neck squamous cell carcinoma (HNSCC) have demonstrated that tumor-derived exosomes support various tumor biological behaviors, whereas the functional roles of stroma-derived exosomes remain largely unknown. Although these exosomes are emerging as promising targets in early diagnosis, prognostic prediction, and pharmaceutical carriers for antitumor therapy, there are still multiple hurdles to be overcome before they can be used in clinical applications. Herein, we systematically summarize the promotive roles of the epithelium and stroma-derived exosomes in HNSCC and highlight the potential clinical applications of exosomes in the treatment of HNSCC.

## 1. Introduction

Head and neck cancer, including cancers in the oral cavity, oropharynx, hypopharynx, nasopharynx, and larynx, is the sixth most common type of cancer, with an estimated 600,000 new cases occurring globally each year [1,2]. Among these, more than 90% are head and neck squamous cell carcinoma (HNSCC) based on their histopathological type [3]. Most HNSCCs arise from stratified epithelia of the skin, oral cavity, and pharynx and in the larynx. Although the multidisciplinary approach using surgical resection with or without radio- and chemotherapy is still the gold standard for treatment, the five-year overall survival rate of HNSCC patients was reported to be merely 60% [4], indicating that novel targeting approaches for improving patient survival and quality of life with HNSCC are required. To date, most of the previous preclinical trials based on altered gene expression in cancer epithelial cells have failed, and one of the potential reasons is the limited understanding of the crosstalk between the cancer epithelium and the tumor microenvironment (TME) in therapeutic resistance. The TME, which is comprised of cancer-associated fibroblasts (CAFs), immune cells, and other stromal cells, plays an important role in tumor progression of HNSCC [5,6]. Since cancer stromal cells play promotive and supportive roles in tumor progression, traditional therapeutic paradigms are often insufficient to exterminate tumor cells. Thus, as we previously concluded [7], targeting the crosstalk between stromal cells and cancer epithelial cells is a promising avenue in anticancer therapy.

Exosomes are small extracellular vehicles (EVs) of endosomal origin with a diameter ranging from 40 to 160 nm that widely engage in the crosstalk between cancer cells and stromal cells [8]. The cargo of exosomes is abundant in proteins, lipids, RNA and DNA, and other compounds. Frequent exosome-guided cellular and molecular communication is reported to promote information transmission in cancers [9,10,11]. In the TME, stromal cells are connective cells including CAFs, mesenchymal stem cells (MSCs), and endothelial cells (ECs) that maintain the structural framework in tumor, and another class of stromal cells that maintains cancer homeostasis is immune cells including dendritic cells, B cells, T cells, macrophages, and natural killer (NK) cells [12]. These stromal cells produce various exosomes involved in tumorigenesis, tumor growth, cancer recurrence, and metastasis due to their versatile roles in promoting cancer cell proliferation, migration, invasion, and therapeutic resistance [13,14,15,16,17]. Therefore, exosomes have become candidates for noninvasive liquid diagnosis and monitoring targeting cancer treatment [18,19]. Herein, we summarize the roles of cancer epithelium/stroma-derived exosomes in mediating tumor progression and their diagnostic and therapeutic applications in HNSCC, which establishes a foundation for advances in exosome-directed early diagnosis, prognostic prediction, and antitumor therapy in HNSCC.

## 2. Biological Characteristics of Exosomes in HNSCC

Exosomes are nanosized vesicles, and those that are secreted by tumor cells are called tumor-derived exosomes (TDEs). The total amount of exosomes released by tumor cells is about 10-fold more than from normal cells [20]. Typically, TDE components include proteins, lipids, DNA, multiple RNA species, and other substances that can be taken up by recipient cells and have the function of regulating receptor cell gene expression, tumorigenesis, angiogenesis, TME reprogramming, and immune tolerance, thereby promoting metastasis and recurrence and therapy resistance [21,22,23]. Recently, various methods, including centrifugation, size exclusion chromatography (SEC), polymer-based precipitation, (immuno-)affinity capture, and microfluidic approaches, have been developed to isolate exosomes. TDEs and stroma-derived exosomes are a highly heterogeneous population of membrane vesicles, varying according to the type of cancer and even stage for the same tumor type, characterized by differences in size, secretion level, morphology, cargo, recipient cells, functional properties, and potential mechanisms (Table 1). TDEs range from 30 to 200 nm in size, whereas stroma-derived exosomes are 40–150 nm. Interestingly, the metastatic OSCC cells secrete larger exosomes than those derived from the parental OSCC cells [24], suggesting that larger size exosomes probably transport more molecular contents for metastasis at the late stage of tumors. Transmission electron microscopy (TEM) is commonly used for exosome visualization. In TEM images, the majority of exosomes secreted by tumor cells and stroma cells exhibit a round or spherical shape. For the cargo in exosomes, protein and miRNA are the two major types of content that have been considerably explored in the context of tumor progression in HNSCC. Previous studies showed that exosomes contain a vast array of proteins, including membrane trafficking and cytoskeletal proteins, signal transducers, and heat-shock proteins, in addition to a high abundance of exosomal microRNAs. The recipient cells of TDEs or stroma-derived exosomes suggest that the exosomes transport cargo from one cell to another in the TME and, thus, can initiate signaling responses for different biological behaviors in HNSCC.

## 3. The Effects of Cancer Epithelium Derived Exosomes on Tumor Progression in HNSCC

### 3.1. Role of Cancer Epithelium Derived Exosomes in Tumorigenesis

**Figure 1:**
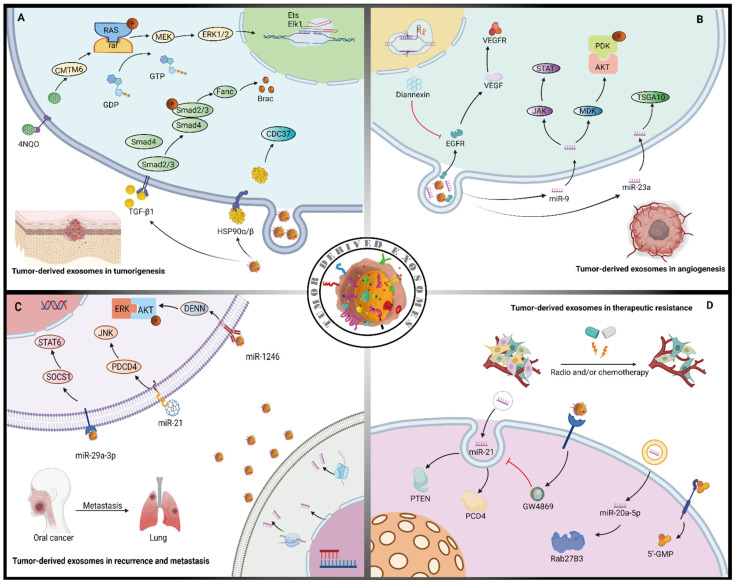
The effects of tumor-derived exosomes in tumor progression. (**A**) Tumor-derived exosomes rich in protein (HSP90α/β,TGF-β1) transfer to adjacent cells to promote tumorigenesis through the immunosuppressive effects. Tumor-derived exosomes CMTM6 induce polarization of M2-like macrophages through the MEK/ERK1/2 signaling pathway. (**B**) Tumor-derived exosomes miRNA (miR-9, miR-23a) and protein (EGFR) raise the formation of new blood vessels during tumor progression via the autocrine VEGF/VEGFR pathway. (**C**) Tumor-derived exosomes miRNA (miR-29a-3p, miR-21, miR-1246), which can be transferred to recipient cells and increase the incidence of recurrence and metastasis of cancer by activating the STAT6, JNK, and ERK signaling pathways, respectively. (**D**) Tumor-derived exosomes miR-21 conveyed to parental cells can be attenuated by the inhibitor (GW4869) promoting chemoresistance. Tumor-derived exosomes miR-20a-5p transfer to adjacent cells leading to radiation tolerance. Back arrows: promotion; red “T” arrows: inhibition.

Tumorigenesis is the process in which normal epithelial cells transform into malignant cancer cells characterized by properties of high proliferation, evasion of apoptosis, immunosurveillance evasion, malignant invasion, and metastasis. A variety of potential mechanisms related to carcinogenesis in HNSCC and TDEs are involved in this process. For instance, Ono et al. provided in vitro evidence showing that metastatic oral cancer-derived exosomes play a role in promoting the epithelial–mesenchymal transition (EMT) process in normal epithelial cells, which is required for tumor initiation [27]. Mechanistically, they found that HSP90-enriched oral metastatic TDEs induce tumor-associated macrophage (TAM) polarization in M2 macrophages, supporting cancer progression, with simultaneous CDC37/HSP90α/β depletion attenuating the metastatic TDEs-driven tumor-initiating activity in oral cancer [27] (Figure 1A). Similarly, Pang et al. found that exosomal CMTM6 secreted by tumor cells induced polarization of M2-like macrophages through the ERK1/2 signaling pathway, thereby promoting malignant progression and participating in mediating crosstalk between cancer cells and macrophages in oral squamous cell carcinoma (OSCC) [42] (Figure 1A). Of note, the CMTM6 levels were positively associated with M2-like macrophage infiltration in the spontaneous OSCC induced by 4-nitroquinoline 1-oxide (4NQO) [42]. In another 4NQO model of carcinogenesis in OSCC, Beatrice et al. showed that application of TDEs isolated from murine or human OSCC cells were sufficient to induce carcinogenesis in mice bearing premalignant lesions by reducing T cell infiltration [33]. Ye et al. have shown that NPC-derived exogenous miRNAs affects T cell proliferation, differentiation and cytokine secretion, which may be related to accumulation of exogenous miRNAs targeting MAPK1 and JAK/STAT pathways [30], indicating the bidirectional effects of exosomes between cancer cells and stromal cells in NPC. Altogether, this evidence suggests that tumor-derived exosomes mainly mediate tumor initiation through crosstalk between cancer cells and immune cells, especially affecting the feedback regulation of TAM polarization and the immunosuppressive effects.

### 3.2. Role of Cancer Epithelium Derived Exosomes in Angiogenesis

Angiogenesis is the formation of new blood vessels from the existing vasculature and it plays a central role in providing oxygen and nutrients to tumors [43]. It has been reported that exosomes are enriched in angiogenesis-related factors, including angiogenic proteins, miRNAs, and mRNAs [44], and accumulating evidence suggests that TDEs promote angiogenesis during tumor progression. Vascular endothelial growth factor (VEGF) is one of the most important angiogenesis-related factors in cancer. For instance, in HNSCC, TDEs containing oncogenic EGFR are taken up by endothelial cells both in vitro and in vivo, activating the autocrine VEGF/VEGFR-2 pathway to induce angiogenesis [45] (Figure 1B). Wang et al. showed that a strong pro-angiogenic effect of TDEs shuttling angiogenin, bFGF, and VEGF could stimulate endothelial cells for angiogenesis by activated STAT3, c-Jun N-terminal kinase (JNK), and p53 signal pathways in vivo [46]. Membrane phosphatidylserine mediates the exchange of TDEs by blocking membrane phosphatidylserine with diannexin as demonstrated in mouse models, and tumors therefore exhibited lower microvascular densities than the controls [45]; Bao et al. also found a relationship between microvascular density and prognosis in nasopharyngeal carcinoma (NPC), indicating that TDE-derived miR-23a promotes angiogenesis by targeting TSGA10, and the high levels of miR-23a in metastatic and premetastatic tissues suggests a close relationships between overall survival time of NPC patients, play a promotive role in angiogenesis [47] (Figure 1B).

In another NPC study, TDE-derived miR-9 inhibited endothelial tube formation by targeting MDK and regulating PDK/AKT signaling pathways [48] (Figure 1B), suggesting that different types of miRNAs in TDEs exert different functions in angiogenesis. Interestingly, in contrast to its role in NPC, Zhang et al. found that TDE-derived miR-9 promotes angiogenesis by activating the JAK-STAT pathway in endothelial cells for various types of cancers, including non-small cell lung cancer, melanoma, pancreatic cancer, and colorectal cancer [49] (Figure 1B). Thus, the effect of TDE-derived miR-9 in angiogenesis appears to be tumor/tissue-specific, and any therapeutic strategies designed to target TDE-derived miR-9 should focus on, or be aware of, its paradoxical roles in the mechanisms of angiogenesis depending on the type of cancer.

### 3.3. Role of Cancer Epithelium Derived Exosomes in Recurrence and Metastasis

In OSCC, about one-third of patients develop local recurrence or distant metastasis after routine treatment [50]. TDEs are reported to play an important role in promoting recurrence and metastasis in HNSCC. For instance, Sakha et al. showed that miR-1246 derived by human oral cancer cells could improve cells’ motility and invasive ability by directly targeting DENN/MADD domain-containing 2D (DENND2D) to activate the ERK and AKT signaling pathways, thereby increasing the incidence of cancer metastasis [34] (Figure 1C). Similarly, Cai et al. found that OSCC-derived exosomes promote M2 macrophage polarization through a mechanism involving the SOCS1/STAT6 signaling pathway in macrophages activated by miR-29a-3p, promoting the invasion of OSCC cells [51] (Figure 1). In esophageal cancer, Liao et al. found that exosomal miR-21 targets programmed cell death 4 (PDCD4) and then activates the downstream c-Jun N-terminal kinase (JNK) cascade, subsequently promoting recurrence and distant metastasis [52] (Figure 1C). Mechanistically, exosome shuttling miR-21 repressed PDCD4 expression by binding to 3′-UTR and increased the expression of MMP-2 and MMP-9 [52]. It suggests that exosome derived miR-21 correlates closely with esophageal cancer metastasis and has the potential to be a biomarker for blocking metastasis. Notably, in HNSCC, one of the most important prognostic determinants is the presence of lymph node metastasis, and lymphatic metastatic spread is correlated with a significant decrease in the patient survival rate. Mouse- and human-derived TDEs may carry microRNA species that change the genetic and protein profiles, thus favoring metastasis formation [53]. In support of this notion, Li et al. found that a hypoxic microenvironment can stimulate OSCC to generate miR-21-rich exosomes in a HIF-1α- and HIF-2α-dependent manner, thereby promoting lymph node metastasis both in vivo and in vitro [36]. Additionally, by HIF-1α or HIF-2α knockdown, exosomal miR-21 levels were significantly decreased in hypoxic conditions [36], indicating that the transcription of exomal miR-21 might be mediated by HIF-1α/HIF-2α. In another study, Ono et al. provided data demonstrating an abundant secretion of HSP90-enriched TDEs in OSCC with lymph node metastasis [24]. Interestingly, by using small interfering RNA to knockdown either HSP90α or HSP90β, there was no apparent effect on the survival of the metastatic OSCC cells; however, the simultaneous knockdown of HSP90α and HSP90β significantly reduced their survival [24], demonstrating that TDE-enclosed HSP90 may be a potential prognostic biomarker and therapeutic target in OSCC with lymph node metastasis. In summary, this evidence suggests that TDEs play a critical role in the recurrence and metastasis of HNSCC by enhancing cancer cell invasion, migration, and survival.

### 3.4. Role of Cancer Epithelium Derived Exosomes in Therapeutic Resistance

Clinically, for those HNSCC patients not undergoing surgical therapy, the addition of chemotherapy, radiation therapy, and/or immunotherapy is necessary for improving their prognosis. Previous studies have suggested that exosomes can induce therapeutic resistance by transferring miRNAs, mRNAs, DNA, proteins, and purine metabolites [54,55]. For instance, Liu et al. demonstrated that TDEs from cisplatin-resistant OSCC cells convey miR-21 to parental cells and induce cisplatin resistance by targeting phosphatase and tensin homolog (PTEN) and PDCD4; the chemoresistance of parental OSCC cells can be attenuated by the inhibitor (GW4869), which suppresses exosome release [37] (Figure 1D), thus suggesting that TDEs may play a role in promoting chemoresistance. For radiotherapy, it has been reported that miR-20a-5p secreted by radiation-resistant nasopharyngeal carcinoma cells is transferred to adjacent cells through exosomes, which inhibits Rab27B by targeting Rab27B3′-UTR (a target of miR-20a-5p), leading to radiation tolerance [56] (Figure 1D). Of note, chemoradiation therapy is already one of the major treatments for HNSCC, though it results in more systemic adverse effects than radiotherapy or chemotherapy alone. Furthermore, Beck et al. found that various exosomes secreted by hypoxic tumor tissues after radiotherapy increase invasion and metastasis in HNSCC, promoting drug resistance during radiotherapy [57] and indicating the need to suppress chemotherapy resistance induced by TDEs during radiotherapy. Additionally, since HNSCC cells escape immunosurveillance for recurrence or metastasis, an in-depth understanding of the potential resistance mechanisms and design of immunotherapeutic approaches is urgently needed. Targeting TDEs may be a promising candidate as another immunotherapeutic avenue. Ludwig et al. performed a clinical study showing that purine metabolites, including adenosine and 5′-GMP in plasma-derived exosomes, increased in patients with early-stage HNSCC with no lymph node metastasis [28], suggesting that immunosuppressive adenosine in the exosomes may play a promotive role in early tumor progression. Interestingly, by comparing the exosome profiles in patients with or without lymph node metastasis, they found that levels of most purine metabolites including adenosine and its derivatives were higher in patients without lymph node metastasis [28]. An alternative hypothesis, we suppose, is that purine metabolites might be mainly used for cell proliferation in the primary site at an early stage for tumor growth while fewer purine metabolites are packaged into exosomes in the circulation for metastatic tumor cells. Therefore, exosome-enclosed purine metabolites may be valuable candidates for a targeted immunotherapeutic approach and sensitive biomarkers for prognostic prediction in HNSCC.

## 4. Cellular Origins of Cancer Stroma Derived Exosomes and Their Roles in HNSCC Tumor Progression

### 4.1. Cancer-Associated Fibroblast-Derived Exosomes in HNSCC

**Figure 2:**
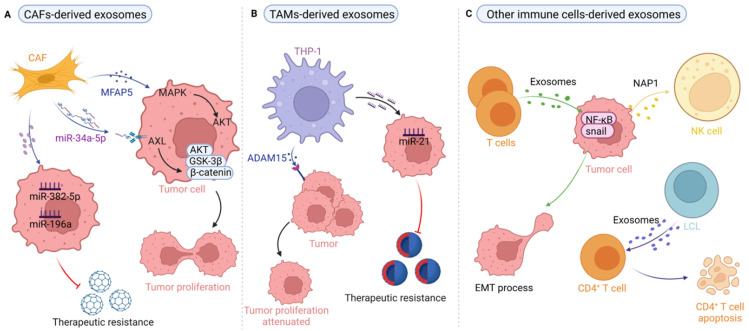
The role of stroma-derived exosomes in tumor progression. (**A**) CAFs-derived exosomes rich in miRNA (miR-382-5p, miR-196a) transferred to tumor cells promote tumor cells migration and invasion, and induce therapeutic resistance. CAFs-derived exosomes miRNA (miR-34a-5p), protein (MFAP5) transfer to tumor cells to facilitate tumor cell proliferation. (**B**) TAMs-derived exosomes miRNA (miR-21) transfer to tumor cells and raise the survival and proliferation of tumor cells with induced therapeutic resistance. TAMs-derived exosomes protein (ADAM15), inhibited tumor proliferation. (**C**) T cell-derived exosomes promote the metastasis of tumor cells by inducing EMT. TDEs NAP1 enhance the cytotoxicity of NK cells. B-cell-derived lymphoblastic-like cell line (LCL) derived exosomes transfer to CD4+ T cell and induce CD4+ T cell apoptosis. Back arrows: promotion; red “T” arrows: inhibition.

We previously systematically summarized the evidence for CAFs, the major cellular component of stromal cells in the TME, which indicated they are critical for cancer occurrence and progression due to their versatile roles [7]. Studies have shown that CAF-derived exosomes shuttle cargo, such as proteins and miRNAs, to perform their various oncogenic functions. For instance, in their proteomic analysis in which they identified 4247 proteins, Principe et al. found that microfibrillar-associated protein 5 (MFAP5) in CAF-derived exosomes promotes proliferation and metastasis of OSCC by activating the MAPK and AKT signaling pathways [58] (Figure 2A). In another study, Li et al. found that the reduced expression of miR-34a-5p in CAF-derived exosomes promotes the proliferation and metastasis of OSCC by binding the downstream target of AXL; furthermore, the miR-34a-5p/AXL axis induces the epithelial–mesenchymal transition (EMT) process for cancer metastasis via the AKT/GSK-3β/β-catenin signal cascade [40] (Figure 2A). Similarly, in OSCC, Sun et al. showed that miR-382-5p was overexpressed in CAFs compared with the fibroblasts from adjacent normal tissue and that CAF-derived exosomal miR-382-5p promotes OSCC cell migration and invasion [59] (Figure 2A). Studies on CAF-mediated therapy resistance have recently reported the use of CAF targeting as an approach for reversing tumor chemoresistance in CAF-mediated anticancer therapy [7,60]. For example, Qin et al. found that exosomal miR-196a secreted by CAFs is resistant to cisplatin and thus enhances cisplatin resistance, and miR-196a could be transferred from CAFs to tumor cells through exosomes, subsequently promoting the survival and proliferation of HNSCC cells [38]. Interestingly, Yeung and colleagues demonstrated that transfer of CAF-derived exosomal miR21 to cancer cells increases chemoresistance to paclitaxel in ovarian cancer cells [61] (Figure 2A). Altogether, the aforementioned evidence suggests that targeting of the miRNAs shuttled in CAF-derived exosomes may be a promising strategy in cancer treatment that addresses issues of resistance, and any targeted strategy based on this strategy should consider tumor/tissue specificity when attempting to optimize therapeutic efficacy.

### 4.2. Tumor-Associated Macrophage-Derived Exosomes in HNSCC

Induced by chemokines and cytokines, among others, TAM polarization can be guided toward the M1 (with pro-inflammatory and antitumor effects) and M2 (with anti-inflammatory and pro-tumor functions) phenotypes to affect tumor growth, migration, and therapeutic resistance in an exosome-dependent manner. For instance, Lee et al. found that macrophages generated from THP-1 induced by phorbol 12-myristate 13-acetate (PMA) are rich in disintegrin and metalloproteinase 15 (ADAM15) and result in delayed tumor growth in vivo, and the tumor volume was also diminished in mice treated with ADAM15-blocked exosomes [62] (Figure 2B), suggesting that ADAM15 exosomes derived from macrophages play a suppressive role in tumor growth and stimulation by PMA for these kinds of macrophages may result in polarization into the M1 phenotype for antitumor effects. Interestingly, Xiao et al. demonstrated that THP-1 treated with exosomes from OSCC cells exhibited an M1-like but not an M2-like phenotype in promoting migration [32], supporting the notion that TAMs are highly heterogeneous stromal cells whose crosstalk with cancer cells is mediated by multiple signals, a point that should be considered in antitumor therapy in OSCC based on the conversion of TAMs from the M2 to M1 phenotype. Of note, the exosomes secreted by THP-1 derived macrophages depends not only on PMA simulation but also the surrounding status including the lipopolysaccharide (LPS) [63] and IL-4 [64,65]. This suggests that the exosomes from THP-1 derived macrophages are highly sensitive to the exogenous stimulus, in part because the exosomes in different cancers exhibit heterogeneity and specificity. Most importantly, macrophage-derived exosomes might reduce the sensitivity of OSCC cells to chemother- apeutic drugs. For example, Tomita et al. found that exosomes derived from the THP-1 and PHM activated Akt/glycogen synthase kinase 3 β signaling pathways to promote proliferation by reducing the proliferation inhibition of 5- FU and CDDP, and that inhibitors of PI3K (LY294002) and Akt (mk-226) may attenuate the decreased exosome-induced chemotherapy sensitivity in OSCC [16]. Similarly, in gastric cancer, TAM-derived-exosome-enclosed miR-21 diminishes chemotherapy sensitivity [14] (Figure 2B). In summary, this evidence indicates that the use of TAM-derived exosomes may be considered a therapeutic approach for anticancer therapy, especially for improving chemosensitivity in HNSCC treatment.

### 4.3. Exosomes Derived from T Cells, B Cells, and NK Cells in HNSCC

Although T cell-derived exosomes have been widely explored in terms of immunity and antitumor effects [66,67,68,69,70], few studies reported on the relation to HNSCC [17]. In esophageal SCC, Min et al. reported that T cell-derived exosomes could promote the metastasis of esophageal cancer cells by inducing EMT through upregulation of β-catenin and the NF-κB/snail pathway [17] (Figure 2C). For B cell-derived exosomes, Saunderson et al. suggested that B cell vesicles were involved in inducing effective cytotoxic T lymphocyte (CTL) responses in DH LMP2A mice [71]. Of note, human B cell-derived lymphoblastic-like cell line (LCL)-derived MHCII^+^FasL^+^ exosomes could induce CD4^+^ T cell apoptosis in an antigen-specific dependent manner and this process might also be mediated by the apoptosis-inducing molecule of FasL [72] (Figure 2C). This evidence suggests that the potential interplay between antigen-specific T and B cell-derived exosomes may contribute to maintaining coordination in tumor immunity. Similarly, there is less evidence related to NK cell-derived exosomes affecting the biological behaviors of HNSCC, while TDEs regulate NK cells through cell–cell communication. For example, Wang et al. found that NAP1 of TDEs enhances the cytotoxicity of NK cells through the IRF-3 pathway [26]. Clinically, Ludwig et al. detected that exosomes in HNSCC patients reduce the expression levels of NKG2D in NK cells [73], indicating the possibility of plasma exosomes as biomarkers for the prognostic prediction of HNSCC. Taken together, these stromal cell-derived exosomes promote tumor progression and attenuate the therapeutic effects by altering the physiological state of recipient cells, indicating that targeting stromal cell-derived exosomes might be an effective strategy for anti-tumor treatment in HNSCC.

## 5. Theranostic Applications of Exosomes in HNSCC

### 5.1. Application of Exosomes in the Diagnosis of HNSCC

Clinically, histopathological and radiological results are widely used in diagnosing HNSCC. However, since the biopsy has inevitable side effects, minimally invasive detection of targeting exosomes in early tumors has become an important research field. CD9, caveolin 1 (CAV1), and tumor rejection antigen 1 (gp96) proteins, which undergo exosomal shuttling, have great diagnostic value in HNSCC [74]. By comparing eight different HNSCC cell lines with normal human oral keratinocytes, Qadir et al. found that the centrosome protein CEP55 is detectable in tumor exosomes but not normal cells [75], indicating that exosomal CEP55 may serve as a potential diagnostic marker for HNSCC. Regarding miRNAs in exosomes, Langevin et al. found that in exosomes isolated from HNSCC patients, the transcription levels of some TDE-enclosed miRNAs, including miR-486-5p, miR-486-3p, and miR-10b-5p, were significantly increased when compared with exosomes isolated from noncancer patients and control cells [76], suggesting that specific TDE-shuttled miRNAs may be valuable diagnostic biomarkers for HNSCC. Recently, such exosomes have been isolated from serum, plasma, and saliva for diagnosing HNSCC (Table 2). In human papilloma virus-16 (HPV-16)-associated oropharyngeal cancer, Kannan and colleagues found that serum exosomal HPV-16-E7 and SIRPA protein levels were higher than in healthy controls [77], illustrating that this approach may be used for early cancer diagnosis. Furthermore, Li et al. found that miR-21 levels were significantly higher in the circulating serum exosomes of OSCC patients than of healthy volunteers [36]. Li et al. demonstrated that PF4V1, CXCL7, F13A1, and ApoA1 from serum exosomes might be related to the metastasis of OSCC, which would help diagnose lymph node metastasis in OSCC [78]. For the plasma-derived exosomes, Schröck et al. demonstrated that SEPT9 and SHOX2 DNA methylation levels of ccfDNA from plasma could be biomarkers for diagnosis in HNSCC patients [79]. Of note, He et al. found a significant increase in salivary exosomal miR-24-3p in 45 preoperative OSCC patients compared with 10 normal controls [80], indicating the potential of salivary exosomal miR-24-3p as a diagnostic biomarker for OSCC. In summary, these results suggest that exosomes and their cargo from serum, plasma, and saliva might be used as biomarkers for the noninvasive diagnosis of HNSCC.

### 5.2. Application of Exosomes in the Prognostic Prediction of HNSCC

Studies have reported that exosomes and exosomal components can be used as biomarkers for cancer prognosis. For example, plasma-derived EBV DNA has been considered a classic biomarker for prognosis and monitoring response in the clinical treatment of HNSCC [89,90]. Previous studies have found that exosomal miR-21 levels were associated with tumor stage and lymph node metastasis in patients with oral, laryngeal, and esophageal SCC [36,91,92]. Similarly, Theodoraki et al. isolated exosomes from the plasma of HNSCC patients and found higher exosomal PD-L1 expression in patients locating in UICC stage III/IV or with positive lymph nodes than those patients who were UICC stage I/II or without lymph node metastasis [93], indicating that the expression of PD-L1 in exosome circulation may serve as a biomarker for predicting tumor prognosis. Other types of exosomal cargo, including epidermal growth factor receptor (EGFR), phosphorylated EGFR, HSP90, and Fas, have been found to have potential as prognostic biomarkers of HNSCC [24,94,95]. Notably, since no single exosomal biomarker is specific enough for use in predicting the prognosis, Li et al. suggested that a combination of CD9, CD63, and HSP70 can be used as predictors of lymph node metastasis in OSCC [78]. The aforementioned evidence indicates that exosomes and their cargo may be promising biomarkers for prognostic prediction, whereas the potential of more specific exosomal markers requires further exploration dependent on improved separation and purification technologies.

### 5.3. Application of Exosomes in the Treatment of HNSCC

TDEs are cell-derived nanoparticles that, due to their low immunogenicity, strong ability to cross physiological barriers, good biological distribution, and bioavailability, play an important role as potential anticancer drug carriers [96]. For example, in NPC, exosomal miR-34c enhances radiosensitivity mainly through inhibiting EMT and targeting β-catenin to suppress the development of malignancy, suggesting that the combination of IR and miR-34c-overexpressing exosomes may be an effective approach for radioresistant patients [97]. In another study, Jiang et al. found that exosomal miR-197-3p not only reduced proliferation and migration of NPC cells, but also reduced tumor growth and radiation resistance of NPC cells by regulating AKT/mTOR phosphorylation activation and HSPA5-mediated autophagy [98]. Similarly, in OSCC, exosomal-mediated miR-30a transfer in regaining sensitivity of cisplatin-resistant cancer cells’ induced apoptosis and autophagy via Beclin1 and Bcl2 regulation [99]. These evidences suggest that some exosomal miRNAs might be the biomarkers of increased overall survival and/or favorable outcome in HNSCC. In head and neck cancer, Concha et al. found that JAK2/STAT1 plays a role in EGFR-mediated immune evasion, and that targeting this signal may be beneficial in blocking PD-L1 upregulation [100]. In oral cancer, Xie et al. confirmed that human bone marrow mesenchymal stem cell-derived exosomes loaded with miR-101-3p regulated MMP2 levels and inhibited migration and invasion of OSCC cells in vitro and in vivo [101], providing a new theoretical basis for treating oral cancer. Additionally, Li et al. found that oxygen pressure in tumor microenvironments regulates myeloid-derived suppressor cell (MDSC) function in an miR-21/PTEN/PD-L1 axis-dependent manner by altering the TDE content to coordinate the antitumor and tumor-promoting γδt cell balance in OSCC [102]. The aforementioned observations demonstrate that exosomes represent bioavailable vehicles that can deliver drugs, proteins, miRNAs, and other molecules to prevent tumor progression and improve therapeutic efficacy via crosstalk between tumor cells and stromal cells.

## 6. Challenges and Future Perspectives

The crosstalk between the epithelium and stroma provides a suitable microenvironment for tumor progression in HNSCC. Exosomes act as mediators of intercellular communication with the advantage of presence in all body fluids, stable biological activity, biological compatibility, diverse sensitivity, and having specific molecules, including proteins and miRNAs, which have effects on many aspects of intercellular substance and signal transduction, including tumorigenesis, development, metastasis, recurrence, treatment of drug resistance, radiation resistance, and immunosuppression in HNSCC. Although TDEs have already been extensively studied in the context of pathogenesis and cancer development, we must broaden our understanding of stroma-derived exosomes and their functional roles in HNSCC. In HNSCC, exosomes have recently emerged as promising and valuable therapeutic targets for antitumor therapy. However, there are still multiple hurdles that must be overcome for the translation of results from bench to clinic. First, a standard method for the separation and purification of exosomes from different liquid samples is urgently needed. Second, the functional mechanisms of exosomes and their contents, especially in the case of stroma-derived exosomes, remain largely unknown. Whether functional exosomes are derived from tumor cells or the host response to the tumor must be determined and confirmed. Third, exosomes have the potential to be used as biomarkers for early diagnosis, nanoscale carriers for antitumor drugs, and tools for prognostic prediction; however, few examples have reached the stage of clinical application. Since exosomes are a highly heterogeneous population of membrane vesicles and no highly specific biomarkers have yet been confirmed to be suitable for use in prediction, an alternative approach combining several biomarkers for preclinical experiments or applications is needed. Overall, information on the specificity, safety, efficacy, and functional properties of exosomes is urgently needed before TDEs and/or stroma-derived exosomes can serve as targeting candidates for anticancer therapy in HNSCC.

## Figures and Tables

**Table 1:** Biological characteristics of exosomes in HNSCC.

Cancer Type	Exosomal Cargo	Concentration	Diameter	TEM Images	Labeling	Donor Cells	Recipient Cells	Mechanism	Activated Pathway	Ref.
Epithelium-derived exosomes										
HNC	CD39/CD73	N/A	N/A	Round	N/A	UMSCC47	Macrophage	Promote angiogenesis	A_2B_R	[25]
HNSCC	NAP1	N/A	103.1 nm	Oval	N/A	CAL 27, SCC25	NK cells	Enhances cytotoxicity of NKcells	IRF-3	[26]
HNSCC	HSP90	50 µg/mL	70–150 nm	Round	N/A	OSCC cell lines	RT7	Initiate EMT	N/A	[27]
HNSCC	Purine metabolites	N/A	80–150 nm	Spherical	N/A	UMSCC47 cells	N/A	Facilitate immune escape	N/A	[28]
NPC	MMP13	N/A	N/A	Round	PKH67	CNE-2	Normoxic CNE2 cells	Enhance metastases	N/A	[29]
NPC	miR-20a-5p	N/A	30–100 nm	Rounded	N/A	NPCTW03 cells	T-cell	Enhance migration and invasion	MARK	[30]
NPC	miR-17-5p	N/A	N/A	Spherical	PKH67	CNE-2	HUVECs	Promote angiogenesis	AKT/VEGF-A	[31]
OSCC	THBS1	N/A	103 nm	Oval	N/A	SCC25, CAL27	Macrophage	Promote migration	p38, Akt, SAPK/JNK	[32]
OSCC	HSP90	N/A	80–111 nm	Round	N/A	HSC-3, HSC-3-M330	HSC-3-M330	Decline metastasis	N/A	[24]
OSCC	PD-L1, FasL	N/A	30–150 nm	Round	N/A	SCCVII, SCC90	T lymphocytes	Promote carcinogenesis	N/A	[33]
OSCC	miR-1246	N/A	30–100 nm	Round	PKH26	HOC313-LM	HOC313-P	Increase migration and invasion	ERK, AKT	[34]
OSCC	miR-210-3p	N/A	30–120 nm	Round	N/A	CAL27	HUVECs	Promote angiogenesis	PI3K/AKT	[35]
OSCC	miR-21	10 μg/mL	50–200 nm	Round	PKH67	OSCC cells	OSCC cells	Increase metastasis	N/A	[36]
OSCC	miR-21	N/A	30–160 nm	Round	DiI	HSC-3-R	OSCC cells	Enhanced drug resistance	DNA damage	[37]
Stromal-derived exosomes										
HNC	miR-196a	N/A	100 mm	Cup shape	DiO	CAFs	CAL 27, SCC-25, HN4	Promotes cisplatin resistance	N/A	[38]
HNC	miR-3188	N/A	30–150 nm	Spherical	N/A	CAFs	HN4	Inhibits tumor growth	N/A	[39]
OSCC	miR-34a-5p	N/A	40–120 nm	Oval	N/A	CAFs	CAL27, SCC15	Promote proliferation metastasis	AKT/GSK-3β/β-catenin	[40]
OSCC	LncRNA LBX1-AS1	N/A	N/A	Round	N/A	Macrophage	SCC-4, CAL-27	Inhibits tumor growth	FOXO3, LBX1-AS1	[41]

HNC: head and neck cancer. NPC: nasopharyngeal carcinoma. HNSCC: head and neck squamous cell carcinoma. OSCC: oral squamous cell carcinoma. ESCC: esophageal squamous cell carcinoma. CAFs: cancer-associated fibroblasts. HSP90: heat shock protein90. MMP13: matrix metalloproteinase-13. HUVECs: human umbilical vein endothelial cells. HOC313-P: HOC313 parent cell line. N/A: not available.

**Table 2:** Theranostic applications of exosomes in HNSCC.

Tumors	Molecules	Cargo	Source	Samples	Isolation Methods	Exosomes Confirmed	Change and Function in the Diseases	Indication	Ref.
HNC	Protein	CD39	Plasma	38	mini-SEC	WB, TEM	Suppressed lymphocyte functions	Cancer biomarkers	[73]
HNSCC	Protein	CD3	Plasma	43	mini-SEC	SEM	Varied during and post ipilimumab therapy	Cancer biomarkers	[81]
HNSCC	Protein	CD45(−)	Plasma	41	mini-SEC	WB, TEM	Higher in high stage compared to low stage	Diagnosis	[82]
HNSCC	Protein	PD-L1	Plasma	17	mini-SEC	WB, TEM	Decreased in therapy and increase at recurrence	Cancer biomarkers	[83]
HNSCC	Protein	CD44v3+	Plasma	44	mini-SEC	WB, TEM	Elevated in stage III/IV and metastases	Cancer biomarkers	[84]
HNSCC	Protein	LOXL2	Serum	36	Ultracentrifugation	WB	Correlated with low-grade HNSCC	Therapy target	[85]
HNSCC	miRNA	miR-486-5p miR-486-3p	Saliva	5	Ultracentrifugation	EB, TEM	Elevated in saliva of cancer	Diagnosis	[76]
HPVOPC	Protein	MUC16 SIRPA	Sera	7	ExoQuickULTRA EV IsolationKit	WB	Augmented invasion and EMT	Diagnosis	[77]
NPC	Protein	BARF1	Serum	50	Ultracentrifugation	WB	Increased in young NPC patients	Diagnosis	[86]
OSCC	miRNA	miR-24-3p	Saliva	49	ExoQuick-TC kit	WB	Maintain the proliferation of OSCC	Diagnosis	[80]
OSCC	miRNA	miR-21	Plasma	108	ExoQuick-TC kit	WB, TEM	Increased migration and invasion of OSCC	Therapy target	[36]
OSCC	miRNA	miR-130a	Plasma	184	ExoQuick ULTRA EV IsolationKit	N/A	Increased and associated with poor prognosis	Diagnosis Prognosis	[87]
OSCC	Protein	PF4V1 CXCL7 F13A1 ApoA1	Serum	20	ExoQuick-TC kit	WB, SEM	Related to the metastasis of OSCC	Diagnosis	[78]
OSCC	miRNA	miR-412-3p miR-512-3p	Saliva	21	Ultracentrifugation	WB, TEM	Up-regulated in cancer	Diagnosis	[88]

HPVOPC: Human papilloma virus-16 (HPV-16) associated oropharyngeal cancer. HNSCC: head and neck carcinoma cells. OSCC: oral squamous cell carcinoma. NPC: nasopharyngeal carcinoma. LSCC: laryngeal squamous cell carcinoma. mini-SEC: mini size-exclusion chromatography. SEM: scanning electron microscope. TEM: transmission electron microscope. LOXL2: lysyl oxidase like 2. WB: western blot. N/A: not available.

## Data Availability

Not applicable.

## Abbreviations

bFGF	basic fibroblastic growth factor
CAFs	cancer-associated fibroblasts
CAV1	caveolin 1
CTL	cytotoxic T lymphocyte
CAFs	cancer-associated fibroblasts
DNA	deoxyribonucleic aid
ECs	endothelial cells
EGFR	epidermal growth factor receptor
EMT	mesenchymal transition
ESCC	esophageal squamous cell carcinoma
ER	endoplasmic reticulum
EVs	extracellular vehicles
HIF-1α	hypoxia inducible factor 1 alpha
HNSCC	head and neck squamous cell carcinoma
HNC	head and neck cancer
HUVECs	human umbilical vein endothelial cells
HSP90	heat shock protein90
LCL	lymphoblastic-like cell line
LSCC	laryngeal squamous cell carcinoma
LOXL2	lysyl oxidase like 2
MDSC	myeloid-derived suppressor cell
MFAP5	microfibrillar-associated protein 5
MSCs	mesenchymal stem cells
MMP13	matrix metalloproteinase-13
mini-SEC	mini size-exclusion chromatography
mRNA	messenger ribonucleic acid
miRNA	micro ribonucleic acid
N/A	not available
NADDP1	nucleosome assembly protein 1
NK	nature killer
NKG2D	natural killer Group 2 member D
NPC	nasopharyngeal carcinoma
4NQO	4-nitroquinoline 1-oxide
OSCC	oral squamous cell carcinoma
PD-L1	programmed death ligand 1
PVOPC	human papilloma virus-16 associated oropharyngeal cancer
PDCD4	programmed cell death 4
PTEN	phosphatase and tensin homolog
RNA	ribonucleic acid
SEC	size exclusion chromatography
TAMs	tumor associated macrophages
TEM	transmission electron microscopy
TDEs	tumor-derived exosomes
TME	tumor microenvironment
VEGF	vascular endothelial growth factor
WB	western blot
